# Dataset of prostate MRI annotated for anatomical zones and cancer

**DOI:** 10.1016/j.dib.2022.108739

**Published:** 2022-11-09

**Authors:** Lisa C. Adams, Marcus R. Makowski, Günther Engel, Maximilian Rattunde, Felix Busch, Patrick Asbach, Stefan M. Niehues, Shankeeth Vinayahalingam, Bram van Ginneken, Geert Litjens, Keno K. Bressem

**Affiliations:** a Charité – Universitätsmedizin Berlin, Corporate Member of Freie Universität Berlin and Humboldt Universität zu Berlin, Institute for Radiology, Hindenburgdamm 30, 12203 Berlin, Germany; b Berlin Institute of Health at Charité – Universitätsmedizin Berlin, Charitéplatz 1, 10117 Berlin, Germany; c Technical University of Munich, Department of Diagnostic and Interventional Radiology, Faculty of Medicine, Ismaninger Str. 22, 81675, Munich, Germany; d Institute for Diagnostic and Interventional Radiology, Georg-August University, Göttingen, Germany; e Radboud University Medical Center, Nijmegen, GA, The Netherlands

**Keywords:** Prostate cancer, Pixel-wise segmentation, T2-weighted imaging, Apparent diffusion coefficient (ADC), Diffusion-weighted imaging, 3.0 Tesla MRI

## Abstract

In the present work, we present a publicly available, expert-segmented representative dataset of 158 3.0 Tesla biparametric MRIs [1]. There is an increasing number of studies investigating prostate and prostate carcinoma segmentation using deep learning (DL) with 3D architectures [2], [3], [4], [5], [6], [7]. The development of robust and data-driven DL models for prostate segmentation and assessment is currently limited by the availability of openly available expert-annotated datasets [8], [9], [10].

The dataset contains 3.0 Tesla MRI images of the prostate of patients with suspected prostate cancer. Patients over 50 years of age who had a 3.0 Tesla MRI scan of the prostate that met PI-RADS version 2.1 technical standards were included. All patients received a subsequent biopsy or surgery so that the MRI diagnosis could be verified/matched with the histopathologic diagnosis. For patients who had undergone multiple MRIs, the last MRI, which was less than six months before biopsy/surgery, was included. All patients were examined at a German university hospital (Charité Universitätsmedizin Berlin) between 02/2016 and 01/2020. All MRI were acquired with two 3.0 Tesla MRI scanners (Siemens VIDA and Skyra, Siemens Healthineers, Erlangen, Germany). Axial T2W sequences and axial diffusion-weighted sequences (DWI) with apparent diffusion coefficient maps (ADC) were included in the data set.

T2W sequences and ADC maps were annotated by two board-certified radiologists with 6 and 8 years of experience, respectively. For T2W sequences, the central gland (central zone and transitional zone) and peripheral zone were segmented. If areas of suspected prostate cancer (PIRADS score of ≥ 4) were identified on examination, they were segmented in both the T2W sequences and ADC maps.

Because restricted diffusion is best seen in DWI images with high b-values, only these images were selected and all images with low b-values were discarded. Data were then anonymized and converted to NIfTI (Neuroimaging Informatics Technology Initiative) format.

**Specifications Table**

**Table:** 

Subject	Radiography and radiology
Specific subject area	Magnetic resonance imaging for diagnosing prostate cancer
Type of data	Image
How the data were acquired	MRI images were acquired with Siemens VIDA and Skyra clinical 3.0 T scanners (Siemens Healthineers, Erlangen, Germany) according to an acquisition protocol that complies with PIRADS version 2.1 guidelines. Examinations contained T2w sequences and diffusion-weighted imaging (DWI) with apparent diffusion coefficient (ADC) maps. T2w sequences were acquired with a slice thickness of 3 mm, no interslice gap, an in-plane resolution of 0.47 × 0.47 mm, a field of view (FOV) of 180 × 180 mm, TE / TR 116 ms / 4040 ms, turbo factor 25, flip angle 160°, and an acquisition time of 3 minutes and 56 seconds. DWI sequences were acquired with a slice thickness of 3 mm, no interslice gap, in-plane resolution of 1.4 × 1.4 mm, field of view (FOV) of 220 × 220 mm, TE / TR 61 ms / 4400 ms, b values of 5, 100, 500, and 1000 s/mm³, total acquisition time 5 minutes and 5 seconds. The software pre-installed on the MRI scanner (version VE11A) was used to calculate an ADC map from b-values of 50 to 1000 s/mm³.
Data format	Filtered
Description of data collection	Men over 50 years who underwent MRI of the prostate at a 3.0 Tesla scanner and then underwent subsequent surgery or biopsy of the prostate were included in this study. All patients were examined at a German university hospital (Charité Universitätsmedizin Berlin) between 02/2016 and 01/2020. The dataset contains 102 patients with histologically verified PCa, and 56 patients who served as controls. Presence of pathologically significant prostate cancer was defined as at least Gleason grade 6 [Gleason Grade Group (GGG) 1] and tumor volume ≥ 0.1 ml. Cancer was confirmed on a patient level.
Data source location	• Institution: Charité - Universitätsmedizin Berlin • City/Town/Region: Berlin • Country: Germany
Data accessibility	Repository name: https://zenodo.org Data identification number: 10.5281/zenodo.6481141 Access at: zenodo.org/record/6481141 Instructions for accessing the data: Please register an account at Zenodo.org, you can then request access to the data. You will need to enter your name and a valid institutional email address. Please note that sharing of the data is prohibited and each person working with the data needs to request access zenodo.org individually. Access to the data will usually be granted within less than 24 hours.
Related research article	L.C. Adams, M.R. Makowski, G. Engel, M. Rattunde, F. Busch, P. Asbach, S.M. Niehues, S. Vinayahalingam, B. van Ginneken, G. Litjens, Prostate158-An expert-annotated 3T MRI dataset and algorithm for prostate cancer detection, Computers in Biology and Medicine (2022) 105817.

**Characteristics of the Patient Cohort**

**Table:** 

	Entire dataset	Training set	Test set
**Numbers**	158	139	19
**Age, years** (± SD, range)	69 ± 9, 35–84	66 ± 9, 35–84	71 ± 10, 53–83
**Verified PCa** (%)	102/139 (73.4)	83/139 (60)	19/19 (100)
**PSA** (IQR)	7.5 (6.3)	7.6 (6.7)	7.4 (7.1)
**PI-RADS scores**: number of patients	4: 55	4: 45	4: 10
	5: 47	5: 38	5: 9
**Gleason grade groups (GGG)**: number of patients	1: 11	1: 9	1: 2
	2: 37	2: 29	2: 8
	3: 24	3: 19	3: 5
	4: 21	4: 18	4: 3
	5: 9	5: 8	5: 1
**BMI, kg/m^2^** (IQR)	25.9 (6.0)	25.8 (6.3)	25.7 (5.9)

## Value of the Data

• Expert annotated MRI datasets of confirmed prostate cancer are rare [8,^12^,13], but are needed to develop sufficient deep learning algorithms to assist radiologists and urologists in the detection and treatment of prostate cancer.
• This dataset provides segmentations from experts in urologic radiology which were extensively reviewed to ensure high quality segmentations. Researchers in computer vision, who do not have access to medical data and/or radiologic expertise, can therefore use it to develop new algorithms for prostate cancer detection.
• The dataset can serve either as a training dataset for developing new algorithms or as a test set for trained algorithms to check their performance and generalizability.
• Part of the dataset remains as a hidden test dataset to be able to check the performance of the trained algorithms on https://prostate158.grand-challenge.org. Researchers can compare trained algorithms under realistic conditions. As the test set remains hidden, overfitting on test data is not possible.

## 1 Objective

The development of robust computational models for prostate imaging is limited by the availability of high-quality datasets for prostate cancer (PCa) and zonal anatomy. Many deep learning papers published in the last years were based on the same open-source patient data from the widely known PROSTATEx challenge dataset, while most studies used non-publicly available single-center datasets. The limited data availability is accompanied by limited availability of open source code. To increase the availability of open data and code for prostate and PCa segmentation, we here provide a comparatively large, publicly available, expert-segmented representative dataset of 158 3T bpMRIs that overall conforms to PI-RADS™ v2.1 guidelines, along with an open-source baseline U-Net algorithm.

## 2 Data Description

The dataset consists of 158 3.0 Tesla MRIs from patients with suspected prostate cancer annotated by two board-certified radiologists. Inclusion criteria for patients were a minimum age over 50 years, an MRI examination with a biparametric imaging protocol meeting PI-RADS v2 technical standards, a subsequent biopsy or surgery, and an available pathology report confirming a diagnosis of prostate cancer. In patients with multiple MRIs, the last examination which was less than six months before pathology was considered. All examinations were performed at a German university hospital (Charité University Hospital Berlin) between 02/2016 and 01/2020. [11]

### 2.1 Structure of the data archive

Folder:
Prostate158_train:
→ train
  → 020
     → adc.nii.gz # ADC sequence in NIfTI format
    → adc_tumor_reader1.nii.gz # Tumor segmentation of reader 1
    → adc_tumor_reader2.nii.gz # Tumor segmentation of reader 2
    → dwi.nii.gz # DWI sequence in NIfTI format
    → t2.nii.gz # T2W sequence in NIfTI format
    → t2_anatomy_reader1.nii.gz # Anatomy segmentation of reader 1
    → t2_tumor_reader1.nii.gz # Tumor segmentation of reader 1
  → 021
  …
  → 158

train.csv # file names with relative paths to be used for training valid.csv # file names with relative paths to be used for model validation during training

**Fig. 1:**
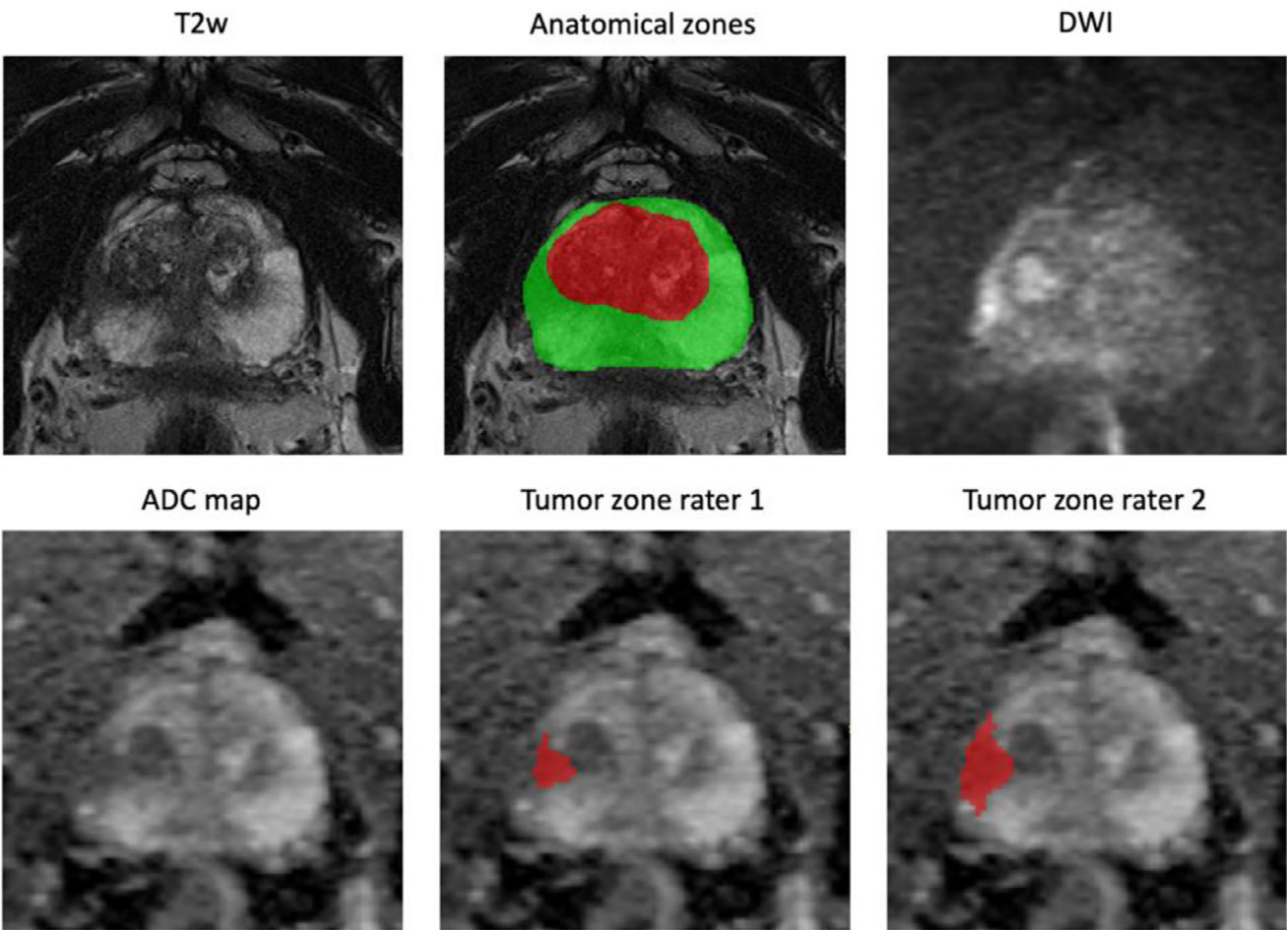
Example axial slices of resampled sequences in the Prostate158 dataset with expert annotations for anatomical zones and tumor zones.

All examinations in the dataset are in axial plane and resampled to the same pixel-spacing and orientation, as well as center-cropped for easier handling. Therefore, the resolution of the original sequences, as given in the below table, is not preserved. Example slices for all sequences with corresponding annotations are provided by Fig. 1.

## 3 Experimental Design, Materials and Methods

### 3.1 Image acquisition and extraction

The data set consists of 158 MRI examinations, all performed on Siemens VIDA and Skyra (Siemens Healthineers, Erlangen, Germany) clinical 3.0-T scanners according to an acquisition protocol that meets current guidelines and using B1 shimming.

Sequence parameters were as follows:

**Table:** 

	T2w	DWI/ADC
Slice Thickness	3 mm	3 mm
Interslice gap	None	None
Inplane resolution	0.47 × 0.47 mm	1.4 × 1.4 mm
FOV size	180 × 180 mm	220 × 200 mm
TE	116 ms	61 ms
TR	4040 ms	4400 ms
Turbo factor	25	-
Flip angle	160°	-
Acquisition times	3:56 min	5:05 min
B-values	-	0, 100, 500, 1000 s/mm3

The ADC map was calculated from b-values ranging from 100 to 1000 s/mm³, using the software pre-installed on the MRI scanner (version VE11A).

Images were then extracted from the PACS and converted to NIfTI (Neuroimaging Informatics Technology Initiative) format for further processing.

### 3.2 Pixel-wise segmentations of anatomical zones and tumor zones

All pixel-wise segmentations were performed with the open-source software ITK-Snap (version 3.8.0; www.itksnap.org). One board-certified radiologist with 6 years of experience segmented the central gland (central zone and transitional zone) and the peripheral zone. Images were then reviewed by a second board-certified radiologist with 8 years of experience.

For prostate carcinoma, both radiologists created independent segmentations of suspected tumor zones. Only lesions with PI-RADS scores of 4 or 5 were included. After a four-week interval, all segmentations were again assessed by the radiologists.

Anatomical zones were segmented in the T2w sequences. Segmentation of PCa lesions was performed in the ADC map and then adjusted in case of over- or underestimation of the tumor extent. This was achieved by creating an overlay of the ADC segmentation mask on T2w and DWI to account for anatomical details and localization of restricted diffusion. Additional segmentations of PCa lesions for T2-weighted images were only performed by reader 1. Segmentations for anatomical zones and tumor zones were saved in different files, each in NIfTI format.

### 3.3 MRI pre-processing

All MRIs were pre-processed using intensity resampling, and centre cropping. In addition, a non-parametric, non-uniform intensity normalization was performed for T2w images to remove distortion fields using the N4ITK algorithm (13). For DWI, only images with the highest b-value (1000) were extracted and the remaining images were discarded.

### 3.4 Resampling

Although the sequences were acquired on the same patient, the FOV, resolution and spacing of the sequences differ. Since each sequence is also manually planned, the orientation of the sequences of a single examination may also be different. For this reason, identical coordinates in the sequences of the same examination do not correspond to the same anatomical region. Therefore, all examinations were resampled so that (1) the size and resolution were identical and (2) the voxel coordinates in different sequences of an MRI corresponded to the same anatomical region. Resampling was performed using SimpleITK 2.0 with the following command:

**import** SimpleITK **as** sitk

reference = sitk.ReadImage(“path/to/reference”)

target = sitk.ReadImage(“path/to/target”)

resampled = sitk.Resample(target, reference)

The T2w sequence was used as reference for all other examinations. For the segmentation mask, the mode for interpolations was set as SimpleITK.sitkNearestNeighbor; for images, the mode was SimpleITK.sitkLinear

### 3.5 Cropping

Prostate MRIs always contain a large part of the pelvis, which is irrelevant for prostate zone segmentation or prostate cancer detection. Because the prostate is reliably located in the center of the examination, images were centered by removing 25% of the image margins along the anterior-posterior and lateral-medial axes.



**Fig. 2:**
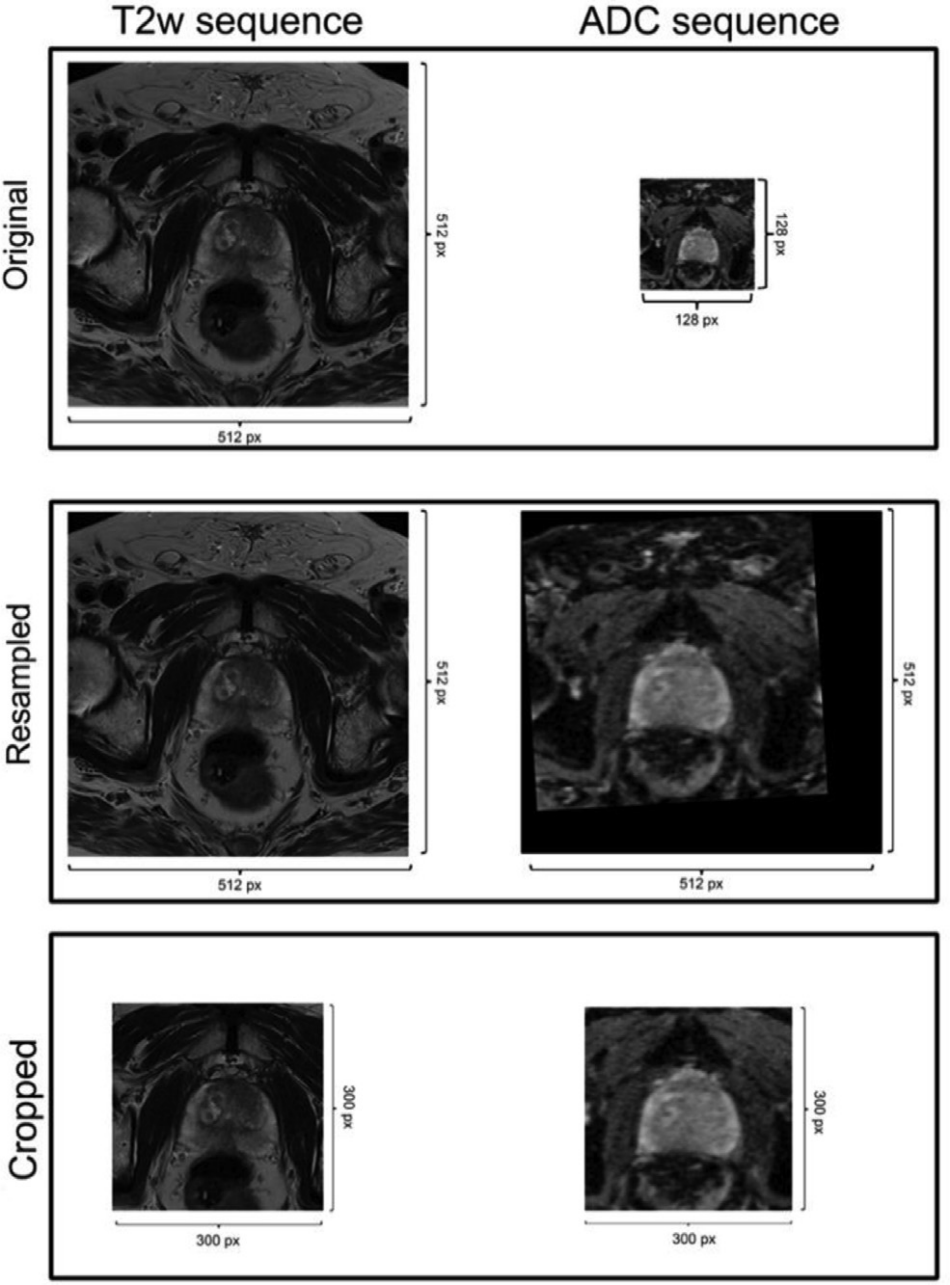
Overview of preprocessing steps taken.

After cropping, the images were saved in NIfTI format. Fig. 2 provides a visual overview of the preprocessing steps.

## Ethics Statements

All contributions to this study were approved by the Institutional Review Board (EA4/062/20), including a waiver of informed consent, and performed in accordance with data protection policy. All research has been carried out in accordance with The Code of Ethics of the World Medical Association (Declaration of Helsinki).

## CRediT authorship contribution statement

**Lisa C. Adams:** Conceptualization, Methodology, Data curation, Investigation, Writing – original draft. **Marcus R. Makowski:** Conceptualization, Methodology, Data curation, Resources, Writing – review & editing. **Günther Engel:** Methodology, Investigation. **Maximilian Rattunde:** Writing – review & editing. **Felix Busch:** Writing – review & editing. **Patrick Asbach:** Writing – review & editing. **Stefan M. Niehues:** Writing – review & editing, Resources. **Shankeeth Vinayahalingam:** Writing – review & editing, Methodology. **Bram van Ginneken:** Writing – review & editing, Methodology. **Geert Litjens:** Writing – review & editing, Methodology. **Keno K. Bressem:** Conceptualization, Methodology, Software, Validation, Visualization, Writing – original draft.

## Declaration of Competing Interest

The authors declare that they have no known competing financial interests or personal relationships that could have appeared to influence the work reported in this paper.

## Data Availability

Prostate158 - Training data (Original data) (Zenodo).
